# Supplementary material for: The BCO2 Genotype and the Expression of BCO1, BCO2, LRAT, and TTPA Genes in the Adipose Tissue and Brain of Rabbits Fed a Diet with Marigold Flower Extract
Source: Int J Mol Sci. 2023 Jan 24;24(3):2304. doi: 10.3390/ijms24032304 (PMC9916731; doi:10.3390/ijms24032304)
Supplement: Supplementary file 1 [file ijms-24-02304-s001.zip › Suplement2.pdf]

Table S1. Ingredients, chemical composition and energy content of the control diet.

Ingredients (%)		Chemical composition and energy content	
Dried alfalfa	33.5	Dry matter (%)	89.15
Oat	17	Crude protein (%)	16.68
Wheat bran	15	Ether extract (%)	3.20
Barley	12.3	Crude ash (%)	6.74
Wheat	9	Neutral detergent fiber (%)	26.63
Soyabean meal	8	Acid detergent fiber (%)	18.57
Linseed	2	Gross energy (MJ/kg)	17.65
Mineral supplements[a]	1.8	Lutein (mg/kg)	21.64
Mineral–vitamin premix[b]	1	Zeaxanthin (mg/kg)	0.14
NaCl	0.4	β-carotene (mg/kg)	9.97
Total	100	Retinol (IU/kg)	9734
		α-tocopherol (mg/kg)	20.71

[a]Mineral supplements: Calcium carbonate, dicalcium phosphate, sodium hydrogen carbonate.
[b]Mineral–vitamin premix (1 kg): vitamin A: 3500000 IU, vitamin D_3: 200000 IU, vitamin E: 28000 mg, vitamin K_3: 200 mg, vitamin B_1: 1500 mg, vitamin B_2: 2800 mg, vitamin B_6: 2800 mg, vitamin B_{12}: 20000 mcg, folic acid: 200 mg, niacin: 10000 mg, biotin: 200000 mcg, calcium pantothenate: 7000 mg, choline: 30000 mg, Fe: 17000 mg, Zn: 2000 mg, Mn: 1000 mg, Cu (copper sulphate x $5H_2O$. 24.5%): 800 mg, Co: 1000 mg, I: 100 mg, methionine: 150 g, Ca: 150 g, P: 100 g.

Table S2. RT-qPCR primers.

Gene	Sequence (5′–3′)	Annealing temperature	Product length	Amplification efficiency
ß-actin	F: CTCCCTGGAGAAGAGCTACG<br>R: TTGAAGGTGGTCTCGTGGAT	59.18°C<br>60.51°C	138 bp	104%
GAPDH	F: TCGGAGTGAACGGATTTGGC<br>R: GCCGTGGGTGGAATCATACT	60.67°C<br>59.82°C	146 bp	105%
BCO1	F: ACGCGACCTCAGAGACAAAT<br>R: TGAAAACGTTTCCAGCAGCG	59.40°C<br>59.97°C	141 bp	102%
BCO2	F: GGCTGTGGTTTTCGGCATTT<br>R: GCTCCTGGTACTGGCACAAA	59.97°C<br>60.25°C	128 bp	89%
LRAT	F: ATGGGCCTGGCATCCTATAC<br>R: CACAGTTGACGTGGGGAAAG	59.00°C<br>59.06°C	93 bp	115%
TTPA	F: CCCAGACATTCTTCCTCTGG<br>R: ATGAATGGGCTCAGAAATGC	59.65°C<br>60.04°C	124 bp	113%

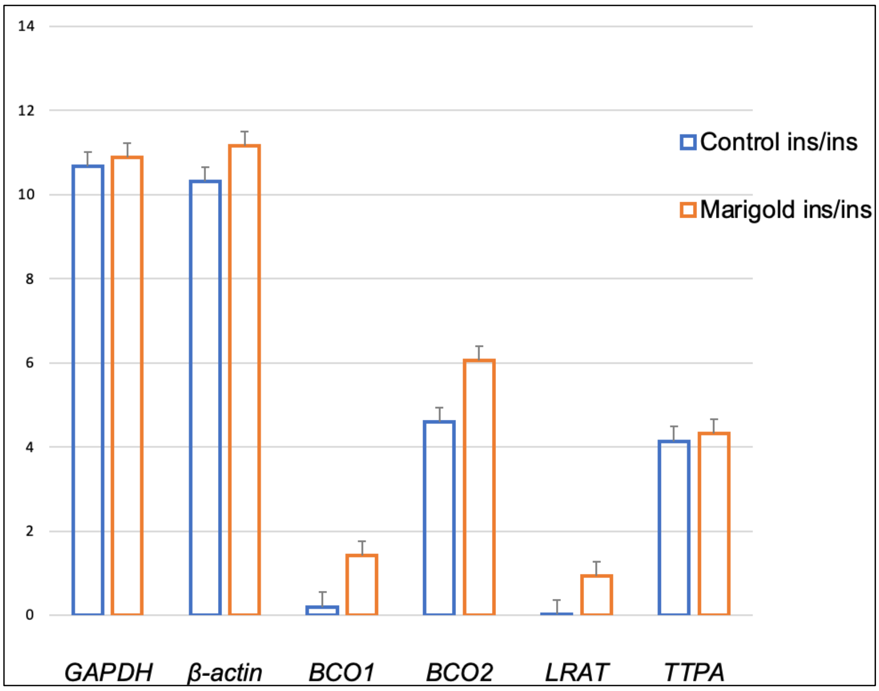

Figure S1. Relative *BCO1*, *BCO2*, *LRAT* and *TTPA* mRNA levels (log2 abundance) in the perirenal fat of rabbits fed different diets (control diet vs. diet with the addition of Aztec marigold flower extract) having ins/ins genotype at codon 248 of the *BCO2* gene. *GAPDH* and *ß-actin* were used as reference genes. Data represent the posterior means (expressed as arbitrary units) ± SEM (standard error of the mean). No statistically significant differences were noted.

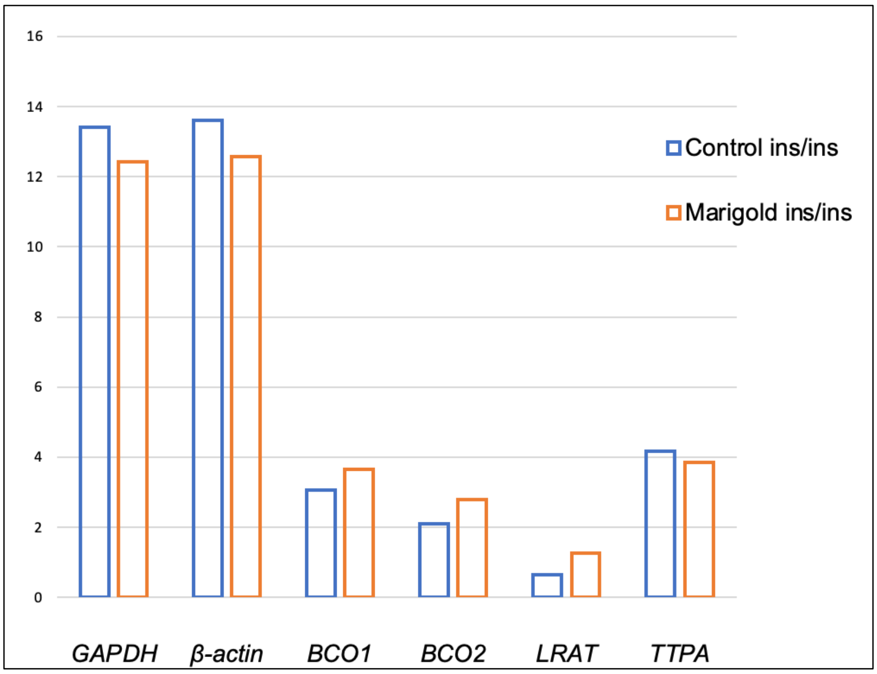

Figure S2. Relative *BCO1*, *BCO2*, *LRAT* and *TTPA* mRNA levels (log2 abundance) in the brain of rabbits fed different diets (control diet vs. diet with the addition of Aztec marigold flower extract) having ins/ins genotype at codon 248 of the *BCO2* gene. *GAPDH* and *ß-actin* were used as reference genes. Data represent the posterior means (expressed as arbitrary units) ± SEM (standard error of the mean). No statistically significant differences were noted.
